# Breast Density Notification: An Australian Perspective

**DOI:** 10.3390/jcm9030681

**Published:** 2020-03-03

**Authors:** Wendy V. Ingman, Bernadette Richards, Jacqueline M. Street, Drew Carter, Mary Rickard, Jennifer Stone, Pallave Dasari

**Affiliations:** 1 Adelaide Medical School Based at The Queen Elizabeth Hospital, University of Adelaide, Adelaide, SA 5011, Australia; pallave.dasari@adelaide.edu.au; 2 Robinson Research Institute, University of Adelaide, Adelaide, SA 5005, Australia; 3 Law School, University of Adelaide, Adelaide, SA 5005, Australia; bernadette.richards@adelaide.edu.au; 4 School of Health and Society, Faculty of Social Sciences, University of Wollongong, Wollongong, NSW 2522, Australia; streetj@uow.edu.au; 5 Adelaide Health Technology Assessment, School of Public Health, University of Adelaide, Adelaide, SA 5005, Australia; drew.carter@adelaide.edu.au; 6 Faculty of Health Sciences, The University of Sydney, Lidcombe, NSW 2141, Australia; mtr2006@bigpond.net.au; 7 Centre for Genetic Origins of Health and Disease, Curtin University and The University of Western Australia, Perth, WA 6009, Australia; jennifer.stone@uwa.edu.au; 8 The RPH Research Foundation, Royal Perth Hospital, Perth, WA 6000, Australia

**Keywords:** breast density, mammographic density, mammogram, breast cancer screening, clinical ethics, health policy, duty of care

## Abstract

Breast density, also known as mammographic density, refers to white and bright regions on a mammogram. Breast density can only be assessed by mammogram and is not related to how breasts look or feel. Therefore, women will only know their breast density if they are notified by the radiologist when they have a mammogram. Breast density affects a woman’s breast cancer risk and the sensitivity of a screening mammogram to detect cancer. Currently, the position of BreastScreen Australia and the Royal Australian and New Zealand College of Radiologists is to not notify women if they have dense breasts. However, patient advocacy organisations are lobbying for policy change. Whether or not to notify women of their breast density is a complex issue and can be framed within the context of both public health ethics and clinical ethics. Central ethical themes associated with breast density notification are equitable care, patient autonomy in decision-making, trust in health professionals, duty of care by the physician, and uncertainties around evidence relating to measurement and clinical management pathways for women with dense breasts. Legal guidance on this issue must be gained from broad legal principles found in the law of negligence and the test of materiality. We conclude a rigid legal framework for breast density notification in Australia would not be appropriate. Instead, a policy framework should be developed through engagement with all stakeholders to understand and take account of multiple perspectives and the values at stake.

## 1. Background

**Figure 1:**
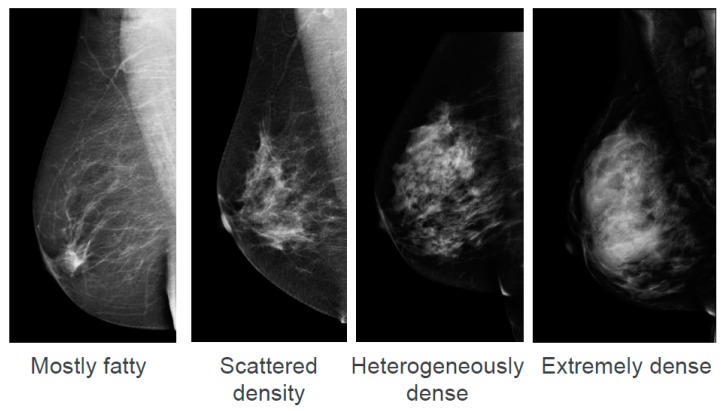
American College of Radiology Breast Imaging Reporting and Data System (BI-RADS) classification system of 4 density categories: “Mostly fatty”, “Scattered density”, “Heterogeneously dense”, and “Extremely dense”. Reproduced with permission from InforMD (www.informd.org.au).

Breast density, also known as mammographic density, refers to white and bright regions on a mammogram. The whiter the mammogram, the denser the tissue appears. The American College of Radiology describe four categories of density in the Breast Imaging-Reporting Data Systems (BI-RADS) Atlas ranging from “Mostly fatty” to “Extremely dense” (Figure 1) [1]. It is estimated that in the U.S.A., 8% of women aged between 40 and 74 years have “Extremely dense” breasts and 35% have “Heterogeneously dense” breasts [2]. These two density categories are often combined and termed “dense breasts”. There is no data on the prevalence of density categories in an Australian population; however, they are likely to be similar to the USA.

The association between breast cancer risk and density was first described by John Wolfe, who reported in 1976 that there appeared to be particular parenchymal patterns observed on a mammogram that were associated with an increased risk of the woman developing breast cancer in the future [3]. However, in the following year, Egan and Mosteller claimed that the finding reported by Wolfe was an artefact of masking—the breast cancer had already been there, just hidden amongst these parenchymal patterns [4]. After 40 years of research and debate, it is now widely accepted that breast density is both an independent risk factor for breast cancer and masks cancer on a mammogram [5,6,7,8]. Together, these two distinct issues result in an increased rate of interval cancers detected in women with dense breasts. An interval cancer is an invasive breast cancer diagnosed in the interval between the completion of a negative screening mammogram and the next screening mammogram. In Australia the interval between screens is 2 years. The interval cancer rate for women with “Extremely dense” breasts is 5.5 per 1000 mammograms, compared to 1.6 per 1000 mammograms for women with “Scattered density” [9].

The current position of BreastScreen Australia and the Royal Australian and New Zealand College of Radiologists is to not notify women of their breast density [10,11]. However, for over 10 years, BreastScreen Western Australia has had the policy of notifying women if they have dense breasts. The information communicated by BreastScreen Western Australia to women with dense breasts is largely focussed on reassuring the woman that dense breasts are normal and common, explaining the lowered sensitivity of the mammogram to detect cancer, and directing women to their GP for a breast check [12].

The increased interval cancer rate that occurs in women with dense breasts has prompted a number of patient advocacy groups in Australia and internationally to call for breast density notification as part of public breast cancer screening programs. In the United States of America, consumer advocacy has led to state, and now federal, legislation mandating that all screened women must be informed of their breast density and the standardisation of breast density reporting laws [13]. The minimum language must include the message that mammograms are significantly less sensitive in diagnosing breast cancer in women with dense breasts, and a reminder to women with dense breasts to consult their doctor if they have any questions or concerns about their breast density. In Australia, both Breast Cancer Network Australia and the Pink Hope charity are lobbying for policy change on breast density [14,15]. In Canada, the patient advocacy group Dense Breasts Canada successfully lobbied for policy change in British Columbia. Breast density notification to all women attending the British Columbia Cancer Breast Screening Program became effective on 15 October 2018 [16], with a number of other Canadian provinces following suit. The screening program in British Columbia does not directly recommend supplementary ultrasounds for women with dense breasts; however, it has provided access to government-funded ultrasounds if women wish to pursue this following consultation with their doctor or nurse practitioner. 

To begin to explore the complex ethical considerations associated with breast density notification, an Australian national stakeholder engagement workshop was conducted, attended by experts in breast density, breast cancer screening, clinical ethics, and the law, as well as patients and clinicians. At the workshop, a range of viewpoints were discussed, and it was clear that whether or not to tell women if they have high breast density, when they have a screening mammogram, is a sensitive and contentious issue. Different stakeholders hold diverse perspectives; for example, some view the issue purely in terms of a right to know and argue that women should have the same information about their own bodies as their doctors have. On the other hand, some caution that knowledge of breast density can cause harm, including anxiety and an increased rate of false positives in patients undergoing supplementary screening, without providing a benefit to population-based breast cancer screening outcomes. The perspectives presented in this manuscript are partially informed by this workshop and through further discussions amongst the inter-disciplinary team of authors. In this perspective, we seek to bring together the diverse views held by different stakeholders, rather than take a position on one side of the debate or the other, and we have not sought to either agree or disagree with current Australian breast density notification policy. Here we identify and discuss the ethical and legal issues associated with breast density notification from an Australian perspective and highlight the key research required to resolve these issues.

## 2. Main Text

Government-funded breast cancer screening is a population-based program guided by the utilitarian objective of maximising the sum total of welfare. However, as breast cancer screening is implemented in a clinic-like situation by clinicians, and women attending the program make decisions for their own benefit, issues related to clinical ethics are also at play [17]. What matters in public health ethics is somewhat different to what matters in clinical ethics, and these perspectives can conflict with each other. Therefore, the framing of this issue within the context of public health ethics or clinical ethics is extremely important. The central ethical themes associated with breast density notification are equitable care, patient autonomy, trust in the doctor–patient relationship, duty of care by the physician, and uncertainties around evidence relating to measurement and clinical management pathways for women with dense breasts. 

### 2.1. Uncertainties in Measurement and Management Pathways

The reluctance amongst clinicians and health authorities to notify women of their breast density is largely related to uncertainties around measurement and clinical management of women with dense breasts. The visual assessment of the BI-RADS scale is very subjective, and the assessment of density can vary amongst radiologists. For this reason, up to 19% of all women screened could potentially be advised differently (dense or non-dense) at the next screen [18], and there is concern that this could lead to unnecessary anxiety and an ineffective approach to supplementary screening. Automated measurement software programs are now becoming available, and use of quantitative measures of breast density, rather than the current qualitative measures, could help address some of the clinical concerns.

There is also uncertainty in what clinical pathway is appropriate for women with dense breasts. There are no randomised-controlled trials evaluating the use of supplemental screening in women with dense breasts that have been run for a sufficient length of time to show a difference in survival. Supplemental breast ultrasounds in women with dense breasts increases cancer detection by 40% [19], and a randomised-controlled trial demonstrates addition of supplementary ultrasounds reduces interval cancer rates and detects cancers of lower grade compared to mammography alone [20]. However, supplementary ultrasounds also increase the rate of recall for further testing (e.g., biopsy) for women who do not have breast cancer, known as a “false positive” [20]. Tomosynthesis (also known as three-dimensional mammography) is a screening modality that increases cancer detection compared to mammography, including in women with dense breasts [21]; however, no randomised-controlled trials have been conducted long enough to investigate improvements in interval cancer-detection rates or survival rates [22]. A randomised-controlled trial demonstrated diagnosis of significantly fewer interval cancers when adjunct magnetic resonance imaging was used for women with extremely dense breasts compared to mammography alone during a 2-year screening period [23]. Follow-up studies will likely investigate whether this is associated with improved survival outcomes. Without studies demonstrating improved survival, the concern is that some of the cancers detected by supplementary screening modalities could result in diagnosis of cancers that are slow-growing, and these cancers would never have threatened the patient’s life (known as “overdiagnosis”).

### 2.2. Equitable Care

BreastScreen Australia is a population-based screening program aimed at reducing breast cancer mortality. For every 1000 women who are screened two yearly from age 50 to 74, eight breast cancer deaths are prevented [24]. We pose the question, “Are women with dense breasts getting the same opportunities for early diagnosis from screening mammography as women with non-dense breasts?” The answer to this question is unclear. Interval cancer rates are higher in women with dense breasts, and interval cancers tend to be more advanced at the time of diagnosis than screen-detected cancers [25]. However, women with “Extremely dense” breasts appear to have a similar survival rate compared to women with breasts of “Scattered density” in the 6 years after a breast cancer diagnosis [26]. In addition, there is insufficient evidence to determine how women with dense breasts could benefit more from breast cancer screening. Therefore, it could be argued that even if women with dense breasts are not receiving the same benefit from screening, any such inequality is not necessarily unjust. Further research is required to determine the long-term benefits of population-level breast cancer screening in women with dense breasts.

From the perspective of clinical ethics, individual women who attend breast cancer screening do so under the belief that it could be of personal benefit to them. Those who then experience interval cancers associated with dense breasts may feel that this belief was false, and that they did not gain any benefit from participating in the program. They may feel poorly served, even misled, by the screening program and that a different pathway ought to have been recommended to them.

Access to supplementary screening for women with dense breasts is not government-funded in Australia. This raises issues with cost and access. Screening modalities such as ultrasound, tomosynthesis, and magnetic resonance imaging are an additional expense and are not widely available in rural areas. However, with the current evidence base, it is not clear whether those who cannot access and afford supplementary screening are at a disadvantage. Research on the cost-effectiveness of supplementary screening for women with dense breasts in an Australian setting would be warranted. 

### 2.3. Supporting Patient Autonomy in Decision-Making

“This is my life and my decision” is a strong theme expressed by patients advocating for breast density notification. The decision to not notify women of their breast density can be viewed as unduly paternalistic, depriving women of involvement in decision-making. On the other hand, providing unhelpful information has the potential to undermine a person’s decision-making and cause worry.

Patients may acknowledge the uncertainties in measurement and management for women with dense breasts but express a strong desire to be part of decision-making despite this. They may consider there to be two different issues at play, one being providing the information and the second being what to do with that information. By contrast, health professionals may view these issues as inter-related. Patients may argue that although providing information might cause some worry, this should not automatically be considered harmful.

There are two distinct risks associated with dense breasts, one being the increased risk of breast cancer and the other being the increased risk that a cancer could be missed on a screening mammogram. Notifying women of these risks may raise different concerns. BreastScreen Western Australia notifies women with dense breasts about the reduced sensitivity of cancer detection by letter, but this letter does not mention the increased risk of breast cancer. There is, however, information about the increased risk of cancer on the organisation’s website. What information women want to know about their own breast density, and how best to notify them, remains unclear, and this could be explored through a deliberative forum. 

### 2.4. Trust in Health Professionals

Women diagnosed with an interval cancer can feel a sense of betrayal when they discover that high breast density may have been a contributing factor to the cancer not being detected on the screening mammogram. As the decision to not notify women of their breast density is a decision made by health authorities, this has the potential to reduce trust in the medical profession. Learning about breast density and its impact occurs at the very time when patients are most vulnerable. They have just been diagnosed with breast cancer and need to rely on the expertise of health professionals for their treatment. So, a loss of trust at this time can be especially damaging.

### 2.5. Duty of Care by the Physician

In Australia, it is uncommon for there to be laws targeting specific legal conditions or diagnoses, therefore guidance must be gained from broad legal principles. The question as to whether or not women should be informed of their breast density has never been addressed by an Australian court and there are no relevant legislative provisions. 

In the absence of specific legislation, the appropriate guidance is to be found in the law of negligence. Here, the questions of duty of care and standard of care are addressed. With regards to duty of care, it is well-established that the doctor–patient relationship is one where a duty of care exists. That duty focusses on the expectation that a doctor will take reasonable care to avoid harm coming to the patient. What is to be considered here is the question of what constitutes “reasonable care”: informing the patient, or not informing them. 

The test for reasonableness differs depending on the characterisation of the action: diagnosis and treatment, or provision of pre-treatment advice. If the action is defined as diagnosis and treatment, then the measure is that of a “responsible body of peers”. Where, however, the action is defined as a failure to inform, which is the most likely here, then the test is one of materiality: would the information have been material to that particular patient? Or, to put it another way, would knowledge of breast density have impacted on the patient’s decision-making processes? The authority for the test of materiality is the seminal case of *Rogers v Whitaker* (1992) 175 CLR 479 where it was concluded that “[t]he law should recognise that a doctor has a duty to warn a patient of a material risk inherent in the proposed treatment; a risk is material if, in the circumstances of the particular case, a reasonable person in the patient’s position, if warned of the risk, would be likely to attach significance to it...”.

The legal test is an objective one based on subjective characteristics. Therefore, the question to be asked would be as follows. Would the reasonable, healthy, asymptomatic woman view this information as material? In answering this question, the context is always significant. For example, if the woman who has decided to have the screening test and who is identified as having dense breasts also has a family history of breast cancer, and expressed concern about it, then the question becomes as follows. Would the reasonable, healthy, asymptomatic woman with a family history of breast cancer and an expressed concern about it view this information as material? It is this lack of certainty that demonstrates the need to maintain the flexible legal test and to supplement it with clear policy guidelines. To lock in a specific, narrowly defined test would either create unnecessary obligations or remove essential protections from some vulnerable women. 

## 3. Conclusions

Breast density notification is an ethically and legally complex issue. It intersects public health ethics and clinical ethics and brings out different perspectives from different stakeholders. Central issues include equitable care, patient autonomy, trust, duty of care, and uncertainties around evidence relating to measurement and clinical management pathways for women with dense breasts. Further research is required, including analysis of long-term health benefits of screening in women with dense breasts, an economic evaluation to determine the cost-effectiveness of supplementary screening, and a deliberative forum to determine what Australian women want to know about their breast density. Legal guidance on this issue must be gained from broad legal principles found in the law of negligence and the test of materiality. A rigid legal framework for breast density notification in Australia would not be appropriate. Instead, a policy framework should be developed through engagement with all stakeholders to understand and take account of multiple perspectives and the values at stake.
